# The Pathology and Treatment of Venereal Diseases

**Published:** 1861-10

**Authors:** 


### BOOKS RECEIVED FOR REVIEW.

*The Pathology and Treatment of Venereal Diseases.—By* FREEMAN J. BUMSTEAD, M. D., *New York.*—BLANCHARD & LEA, *Philadelphia, Publishers.*

A well written book, which clearly and ably states all that is known upon a given subject, is always very welcome, even if it gives us nothing with which we were not already familiar. But a book which, besides merits of style and method, contains much that is new, is a valuable acquisition.

The volume under consideration is really what it was intended to be—" a full and comprehensive treatise upon venereal diseases"—including the results of recent investigations, which add so much to our knowledge upon the subject. These additions to our knowledge are in the highest degree important, and not only render our views of the subject more definite and satisfactory, but completely overthrow many of our previous conclusions.

After a careful reading of this book, the conviction is irresistible, that much of our reasoning and practice, as far as at least one of these diseases is concerned, has been entirely founded in error. What we have been in the habit of considering as well established fact, proves to be no fact at all.

In order to be convinced how far this is true, we have but to compare what we have hitherto believed well ascertained, with what we now find to be very satisfactorily proved. Let us look at the important and surprising discoveries which the author makes known to us. First of all, the division of chancres into two classes—the simple or non-infecting, and the infecting chancre; the former never, the latter invariably followed by general syphilis—the former purely local, the latter in only slight degree so, but rather a mark of general constitutional infection. These statements sufficiently startling, do not contain all that is new upon the subject. We learn further, that secondary lesions are contagious, and that the result of the infection is equally with that of the primary, a chancre—that the infecting chancre cannot be inoculated upon the person bearing it, as a rule, though not perhaps without exception, and, therefore, one attack confers, generally, an immunity against a second—that it has a distinct period of incubation, and destruction has no effect to prevent general infection—that syphilis has its laws, which, like those governing variola, measles, &c., produce a certain degree of regularity in the appearance of its manifestations.

A very little attention to the above statements cannot fail to satisfy us that we have had no really accurate knowledge of the true nature of syphilis —for it is probable that the number of surgeons is very small who have not, all their days, treated the non-infecting chancre as if it were the true syphilitic sore, and who have not had entire faith in the abortive treatment, if resorted to sufficiently early, as applied to the infecting chancre.

In the first part of his book, the author considers gonorrhœa and its complications. Of this it is enough to say, that it treats its subject in a very satisfactory manner. It seems quite clear that urethritis of great severity is not always of a specific origin.

The second part is devoted to the consideration of the Chancroid, its complications, and syphilis. It is hardly necessary to state that the chancroid is the non-infecting chancre, and like itch, favus and gonorrhœa, is a contagious, but in no sense a constitutional disease. Its occurrence, moreover, gives no immunity against a second attack. On the other hand, the true chancre is but the beginning, or as the author expresses it, the "initiatory lesion" of syphilis already acquired, which will in due time be followed by its general manifestations, and confers a certain degree of immunity against its second occurrence. We must give up our chancre-type, viz: the "excavated sore with sharp edges," and watch with eager and suspicious eyes, "slight erosions," with a tendency to induration, for the former will most probably prove a chancroid, while the latter may, usher in that terrible malady syphilis, which, by being better known, is more thoroughly to be dreaded. Upon the whole, though the author has shown us the dreadful nature of the syphilitic virus more clearly than we ever before felt, yet there is much comfort to be derived from it. It explains away many of the difficulties of our experience, enables us to expect more definite results from practice, and puts some limits to our notions of the syphilitic diathesis.

We feel much indebted to the author, in his remarks on treatment, for the statement that syphilis, in some degree, tends to self limitation, and not inevitably to death; and that in cases which pursue a disastrous course in spite of treament, we are not altogether without hope.

The author gives a most decided quietus to abortive treatment, though in place of it, as far as local effect is concerned, he favors destructive treatment by caustics.

In describing his methods of treatment, he has given sufficient attention to detail to give a practical value to his work, keeping in mind, that the success of remedies depends as much on their judicious use, as on their intrinsic value. In regard to the new doctrine of syphilization, there appears to be reasonable ground for believing, as the author states, that the treatment of syphilis by syphilization, "in efficiency and safety, is equal, and probably superior, to the treatment of the same disease by mercury." This only considers its application to a constitution already contaminated by the syphilitic poison; and however repugnant to our feeling, if true, we must not refuse to follow where truth leads. We could willingly say more, if thereby we could more convincingly impress upon our readers a sense of the great merit and value of Dr. Bumstead's work; but we cannot close without saying that, not only do we consider his book the best on the subject, as far as our knowledge extends, but it appears to us the *only* one which can guide to a correct practice—the recognition of the duality of the chancrous virus being its only foundation. No surgeon should undertake the treatment of the syphilitic disease, without a full knowledge of the recent views upon the subject, and for this purpose he will find no more valuable source than the work of Dr. Bumstead.

The book itself, in its appearance and execution, is every way very creditable to the publishers; and it gives us pleasure to commend the great improvement in the general appearance of medical books which have lately been issued by the publishers of this work. L.

